# Metal ion levels in large-diameter total hip and resurfacing hip arthroplasty-Preliminary results of a prospective five year study after two years of follow-up

**DOI:** 10.1186/1471-2474-13-56

**Published:** 2012-04-11

**Authors:** W Maurer-Ertl, J Friesenbichler, P Sadoghi, M Pechmann, M Trennheuser, A Leithner

**Affiliations:** 1 Department of Orthopaedic Surgery, Medical University of Graz, Graz, Austria

**Keywords:** Hip resurfacing, Total hip arthroplasty, Prosthetic wear, Cobalt, Chromium

## Abstract

**Background:**

Metal-on-metal hip resurfacing is an alternative to metal-on-metal total hip arthroplasty, especially for young and physically active patients. However, wear which might be detected by increased serum ion levels is a matter of concern.

**Methods:**

The aims of this preliminary study were to determine the raise of metal ion levels at 2-years follow-up in a prospective setting and to evaluate differences between patients with either resurfacing or total hip arthroplasty. Furthermore we investigated if the inclination of the acetabular component and the arc of cover would influence these findings. Therefore, 36 patients were followed prospectively.

**Results:**

The results showed increments for Co and Cr in both implant groups. Patients treated with large-diameter total hip arthroplasty showed fourfold and threefold, respectively, higher levels for Co and Cr compared to the resurfacing group (Co: p < 0,001 and Cr: p = 0,005). Nevertheless, we observed no significant correlation between serum ion levels, inclination and arc of cover.

**Discussion:**

In order to clarify the biologic effects of ion dissemination and to identify risks concerning long-term toxicity of metals, the exposure should be monitored carefully. Therefore, long-term studies have to be done to determine adverse effects of Co and Cr following metal-on-metal hip replacement.

## Background

Metal-on-metal hip resurfacing or total hip arthroplasty (THA) became an accepted and widespread procedure for joint replacement due to the favourable wear pattern, especially in young and physically active patients [1-5]. Therefore, several manufacturers introduced different hip resurfacing systems and large-diameter hip arthroplasty devices.

Nevertheless, the number of revisions for failed hip arthroplasties using metal-on-metal articulation increased, especially following hip resurfacing. The main drawback of metal articulation is the production of metal wear debris leading to elevated concentrations of metal ions (Cobalt (Co), Chromium (Cr) and Molybdenum (Mo)) within the hip joint, the periarticular soft tissues and systemically [3,6-20]. Furthermore, there are several other factors leading to a variability in the serum levels of metal ions such as different implant size, incorrect adjustment of the components (inclination, anteversion, arc of cover), and differences in manufacture and metallurgy as well as metal corrosion [4-8,16,17,19,21,22].

Up to now there have been many studies investigating the characteristics of metal ions in the human body after hip arthroplasty with metal-on-metal bearings [1-18,21-28]. Increased concentrations have been found to be associated with hypersensitivity, local soft tissue reactions (pseudotumors), metallosis, and several other adverse effects [1-3,5-7,11,18-20,23,25]. Nevertheless, it remains unclear whether adverse reactions are dose-dependent and whether they are mediated by an immune response or if they are a toxicological effect [6].

Herein, we present the preliminary results of serial serum metal ion determination after 2 years in a prospective series over a five year period in patients who had undergone metal-on-metal hip resurfacing or large-diameter total hip arthroplasty. The primary aim of this preliminary study was to determine the raise of metal ion levels at 2-years follow-up in a prospective setting. The secondary aim was to evaluate differences between patients with either resurfacing or total hip arthroplasty and if the inclination of the acetabular component and the arc of cover would influence these findings.

The first study hypothesis was, that metal ion levels would increase after 2-years of follow-up in both groups. The second hypothesis was that inclination of the acetabular component and the arc of cover would positively influence elevated metal ion levels.

## Methods

A series of 36 patients who underwent metal-on-metal THA or hip resurfacing were followed prospectively over a 5-year period. For resurfacing hip arthroplasty a porous coated anatomical system was used (Articular Surface Replacement System-ASR^TM^, DePuy, Warsaw, IN). For metal-on-metal THA a porous coated anatomical system with a large diameter femoral head (both ASR^TM^ XL Head, DePuy) was combined with a standard femoral shaft (Corail® or Future®, DePuy). All prostheses were manufactured from Co-Cr-Mo alloy according to ISO 5832-4.

The surgeries were performed by six senior orthopaedic surgeons, using a modified antero-lateral (THA) or a posterior approach (resurfacing arthroplasty). Press fit fixation of the acetabular components and femoral stems was performed in all patients and the femoral resurfacing component was cemented.

Five men and 3 women were included in the resurfacing group (n = 8 resurfacings). These patients were chosen for this procedure due to their physically activeness and their young age at time of operation (mean: 47-years, range, 33-57 years). The THA group included 28 patients (15 men and 13 women) with a mean age at operation of 52-years (range, 40-61 years). During the study period 4 patients of the THA group required replacement of the contralateral hip (n = 32 THAs) using the same metal-on-metal device. Patient demographics are reported in Table1.

**Table 1:** **Patient demographics, gender distribution and component details**

**Demographics**	**ASR Resurfacing**	**ASR XL Head**
Number of patients	8	28
Number of hips	8	32
M:F (% female)	5:3 (37,5)	15:13 (46,4)
Mean age in yrs (range)	47 (33 to 57)	52 (40 to 61)
Mean body mass index in kg/m^2^	24,5 (21,3 to 27,7)	27,8 (21,6 to 37,6)
Mean acetabular size in mm	55 (50 to 58)	52 (46 to 58)
Mean femoral size in mm	49 (45 to 51)	46 (41 to 51)

All values are mean and shown with their range.

Determination of metal ion concentrations was performed preoperatively, 6 weeks, 3 and 6 months postoperatively, and annually thereafter.

### Metal ion sampling

Blood was taken from 36 patients treated with metal-on-metal hip resurfacing or metal-on-metal total hip arthroplasty using stainless-steel needles attached to 9 mL no additive plastic vacuum tubes (VACUETTE®). All needles and tubes were used from the same batch. None of the patients had a history of renal impairment.

All specimens were centrifuged for 10 minutes at 4300 rpm within 2 hours and stored at 4°C until analysis. The concentrations of Co, Cr and Mo were determined using electrothermal graphite furnace atomic absorption spectrometry (ET ASS) in the same laboratory (Medical and Chemical Laboratory Diagnostic Lorenz & Petek GmbH). This method of analysis has been chosen due to high sensitivity and reduced matrix effects (Zeeman Effect). A second tube was stored at the department at -20°C due to the availability in case of further studies. Furthermore, the metal ion measurements can be repeated at any time if necessary.

For analysis, 300 μl of each serum sample were diluted with 50 μl Modifier und 550 μl Aqua dest. (Rotipuran). Control samples as well as a standard samples were diluted in the same way. Fifty μl Modifier and 850 μl Aqua dest. (Rotipuran) were used as blank values of the reagents. The ET ASS has been repeated twice for each sample and the levels of metal ions in the serum were recorded in concentrations expressed as μg/dl.

The sample to be examined was evaporated in an atomization apparatus and transformed into atomic condition. With graphite furnace atomic absorption spectrometry, solved samples were charged in a graphite tube with a micro pipette and liberated from the solvent and other concomitant agents by stepwise heating before being atomized. This produced a signal with an area proportional to the element of interest. The concentration of the dilution could be calculated by using the dosed volume of the sample.

### Radiological analysis

**Figure 1:**
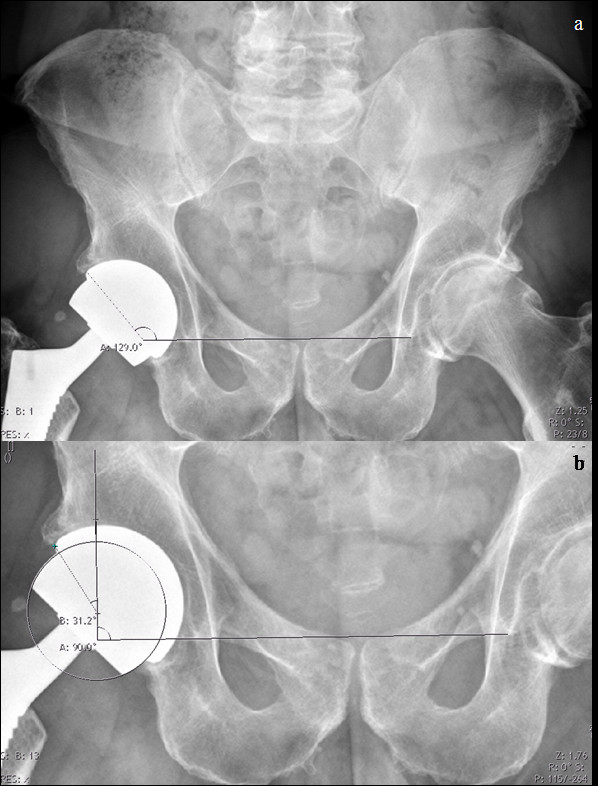
**a&b. Plain radiographs of the pelvis of a 46-year-old female patient with an ASR**^**TM**^**XL Head device two years following implantation.a**) Measuring cup inclination. **b**) Measurements to determine the arc of cover according to the method described by De Haan et al. in 2008.

Weight-bearing, anteroposterior digital radiographs from the pelvis and the operated hip were made one week, 3 months, 1 year and 2 years postoperatively. The inclination angle of the acetabular component was measured from the intersection of two lines on the anteroposterior radiograph of the pelvis, one line across the edges of the component and the other joining both tear trop signs (Figure 1a). According to the study of De Haan et al. [22] the inclination was considered steep if the angle was higher than 55°. Furthermore, the implants' arc of cover was calculated (Figure 1b) [22]. If the arc of cover was less than 10 mm, elevated metal ion levels in serum were expected because the smaller the arc of cover the greater the tendency to rim-loading [6]. All measurements were performed using the Synedra view program, personal version 1.0.12.3 (Synedra information technologies GmbH, Innsbruck, Austria). We correlated metal ion levels with inclination and the arc of cover.

### Statistical analysis

Spearmen's rank correlation coefficient was calculated separated for each implant group to show if there was a correlation between the concentrations of Co, Cr and Mo in the serum, the inclination of the acetabular component and the arc of cover. A *Mann-Whitney-U test* was performed to determine differences in median serum metal ion concentrations at the follow-up checks. This non-parametric test was chosen due to the asymmetric distribution of metal ion levels. A *p-value* of <0,05 was considered to be statistical significant. For statistic analysis the PASW Statistics 16.0 program (SPSS Inc., Chicago, IL) was used.

The study was approved by the Ethics Committee of the Medical University of Graz (EK17-265 ex 05/06) and written informed consent was obtained from all patients.

## Results

**Figure 2:**
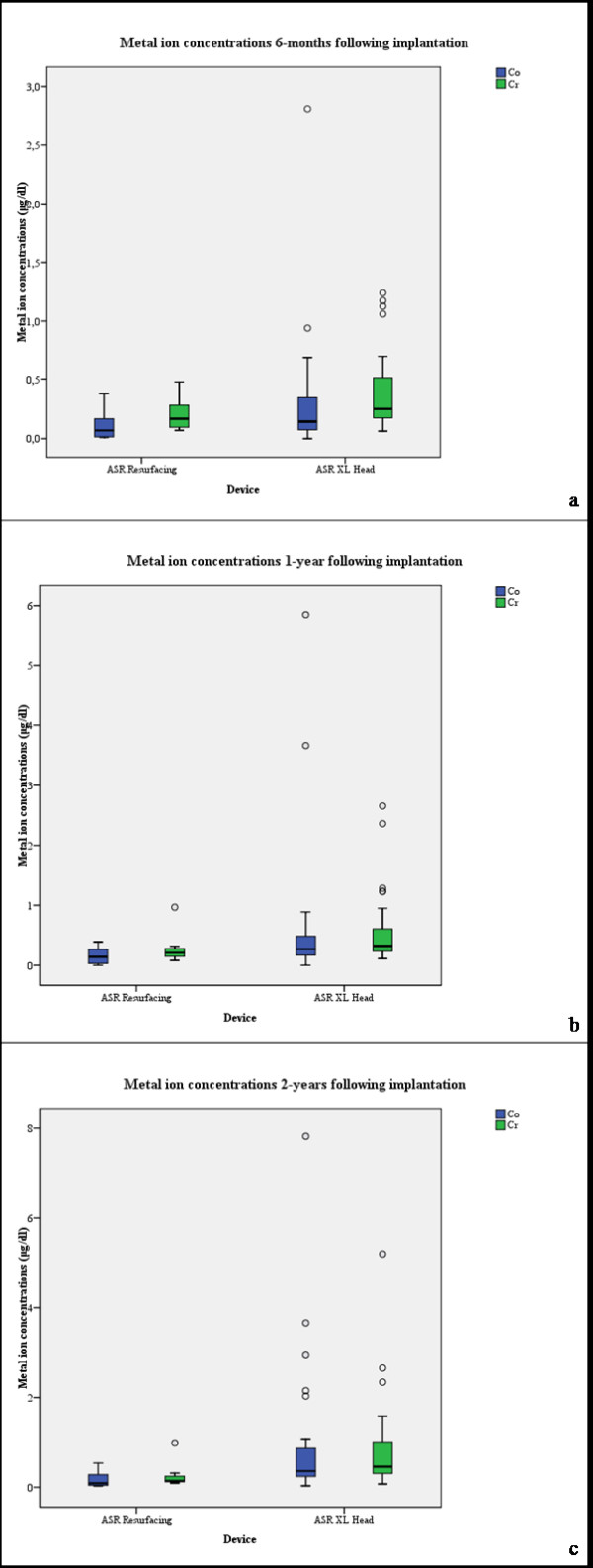
a-c. Box plot of metal ion concentrations devided by resurfacing and THA group, a) 6-months, b) one year and c) two yeras following implantation.

The median results for cobalt and chromium in the serum of patients with the ASR^TM^ resurfacing device were 0,09 μg/dl (range, 0,03-0,54 μg/dl) and 0,141 μg/dl (range, 0,092-0,990 μg/dl) 2 years following implantation (Figure 2a-c; Table2). In comparison to that, patients with the ASR^TM^ XL Head device revealed median concentrations of 0,36 μg/dl (range, 0,03-7,82 μg/dl) and 0,458 μg/dl (range, 0,071-5,198 μg/dl) for Co and Cr (Figure 2a-c; Table2). The values for Co and Cr were fourfold and threefold, respectively, higher in the THA group compared to the resurfacing group. These differences for Co (p < 0,001) and Cr (p = 0,005) were statistically significant (Table 2).

**Table 2:** Preoperative and postoperative median Co and Cr concentrations (range) following metal-on-metal hip arthroplasty

**Serum metal ion levels**	**ASR Resurfacing**	**ASR XL Head**	**Significance**
*Median Co(μg/dl)*			
preoperative	0,02 (0 to 0,03)	0,00 (0 to 0,54)	0,504
6 weeks	0,06 (0,01 to 0,19)	0,07 (0 to 0,30)	0,780
3 months	0,04 (0 to 0,33)	0,10 (0 to 0,66)	0,143
6 months	0,07 (0,01 to 0,38)	0,14 (0,00 to 2,81)	0,134
1 year	0,14 (0 to 0,39)	0,27 (0 to 5,85)	0,068
2 years	0,09 (0,03 to 0,54)	0,36 (0,03 to 7,82)	0,011
*Median Cr (μg/dl)*			
preoperative	0,020 (0,010 to 0,030)	0,026 (0,007 to 0,753)	0,293
6 weeks	0,100 (0,060 to 0,140)	0,101 (0,050 to 0,443)	0,802
3 months	0,137 (0,097 to 0,398)	0,176 (0,059 to 0,724)	0,404
6 months	0,170 (0,070 to 0,475)	0,253 (0,064 to 1,240)	0,099
1 year	0,207 (0,081 to 0,968)	0,324 (0,111 to 2,656)	0,051
2 years	0,141 (0,092 to 0,990)	0,458 (0,071 to 5,198)	0,005
*Inclination in °*			
1 week	43,8 (35,1 to 60,0)	43,6 (30,4 to 60,3)	0,892
3 months	40,9 (35,4 to 61,2)	44,1 (30,7 to 61,3)	0,465
1 year	40,9 (34,9 to 59,3)	44,5 (34,4 to 60,4)	0,465
2 years	41,0 (35,0 to 61,0)	45,5 (28,0 to 68,0)	0,351
*Arc of cover in mm*			
1 week	14,6 (7,7 to 19,2)	13,4 (5,4 to 19,9)	0,372
3 months	15,5 (7,0 to 19,0)	13,9 (5,2 to 19,9)	0,244
1 year	15,2 (8,2 to 19,1)	13,1 (5,7 to 17,9)	0,223
2 years	16,0 (7,6 to 19,5)	13,8 (6,0 to 20,2)	0,281

Mean values with their range are given for the inclination and the arc of cover.

Determining the correlation between the concentration of Co and Cr in the resurfacing group revealed a statistically not significant result (Spearmen: 0,695; p = 0,056), while this correlation was significant in the THA group (Spearmen: 0,805; p < 0,001; Table3).

**Table 3:** Results of correlation analysis

**ASR Resurfacing**				
Follow-up: 1 year	Cr	Co	Inclination	Arc of cover
Co	Spearmen: ,855; p = 0,007		Spearmen: -,229; p = 0,586	Spearmen: ,229; p = 0,586
Cr		Spearmen: ,855; p = 0,007	Spearmen: -,048; p = 0,911	Spearmen: ,048; p = 0,911
Follow-up: 2 years	Cr	Co	Inclination	Arc of cover
Co	Spearmen: ,695; p = 0,056		Spearmen: -,530; p = 0,177	Spearmen: ,539; p = 0,168
Cr		Spearmen: ,695; p = 0,056	Spearmen: -,778; p = 0,023	Spearmen: ,476; p = 0,233
**ASR XL Head**				
Follow-up: 1 year	Cr	Co	Inclination	Arc of cover
Co	Spearmen: ,819; p < 0,001		Spearmen: ,192; p = 0,357	Spearmen: -,176; p = 0,389
Cr		Spearmen: ,819; p < 0,001	Spearmen: ,103; p = 0,623	Spearmen: -,107; p = 0,601
Follow-up: 2 years	Cr	Co	Inclination	Arc of cover
Co	Spearmen: ,805; p < 0,001		Spearmen: ,240; p = 0,201	Spearmen: -,326; p = 0,073
Cr		Spearmen: ,805; p < 0,001	Spearmen: ,234; p = 0,213	Spearmen: -,258; p = 0,161

Spearmen's correlation coefficient was calculated to determine correlations between metal ion concentration, inclination and arc of cover at, one and two years postoperatively.

Two years following implantation, the mean inclination of the acetabular component was 41° (range, 35°-61°) in the resurfacing group and 45,5° (range, 28°-68°) in the THA group (Table 2). Nevertheless, differences in inclination were statistically not significant (p = 0,351). Consequently, there were not significant differences in the arc of cover (p = 0,281; Table2). Calculations revealed no significant correlations between the concentrations of metal ions in the serum, the inclination of the acetabular component, as well as the arc of cover in both groups (Table 3). Probably, the current study failed to show a correlation between metal ion levels, inclination and arc of cover due to the small number of patients included.

## Discussion

The primary aim of this preliminary study was to determine the raise of metal ion levels at 2-years follow-up in a prospective setting. The secondary aim was to evaluate differences between patients with either resurfacing or total hip arthroplasty and if the inclination of the acetabular component and the arc of cover would influence these findings.

The first study hypothesis was, that metal ion levels would increase after 2-years of follow-up in both groups. The second hypothesis was that inclination of the acetabular component and the arc of cover would positively influence elevated metal ion levels.

Our findings showed an increment of Co and Cr concentrations in the serum of patients following metal-on-metal hip resurfacing as well as THA within the first 2-years following implantation with significant differences in Co and Cr concentrations between the resurfacing and large-diameter THA group (Co: p < 0,011, Cr: p < 0,005) 2-years after implantation (Figure 2a-c) and therefore, we confirm the first hypothesis. On the other hand, we refute the second study hypothesis as there was no significant correlation between the inclination of the acetabular component and the arc of cover with metal ion concentration neither in the resurfacing nor in the THA group (Table 3).

Several authors demonstrated that the concentrations of Co and Cr ions increase within the first 3 months to 2 years following metal-on-metal hip arthroplasty (so called run-in period) [3,8,10-14,24,25]. Imanishi et al. [8] related a not significant additional increasement of serum metal ion levels at one year follow-up, whilest Back et al.[10] and Daniel et al. [11,15] observed a decreasing trend within the first 6-years. Sauvè et al. [9] reported threefold and fivefold higher serum concentrations of Co and Cr following metal-on-metal hip arthroplasty compared to a control group during a 30-year follow-up. In 2010, deSouza et al. [2] related their 10-year results following hip resurfacing with two key findings. They found a second increment of metal ion concentrations, five years following hip resurfacing and second they found no significant differences between women and men, which is contradict to other data in the literature. In the present study, we can not provide further information whether the metal ion levels will show a further increasement, whether they will reach a plateau phase or whether they will decrease. It seems that the patients are still in the prosthesis' run-in period and further follow-up has to be done to observe the development of metal ion concentrations.

Matthies et al. [1] recently showed that metal-on-metal hip resurfacing and modular total hip replacements have same wear rates and both types of devices are associated with increased blood levels of metal ions. Therefore, this group suggested that the problems related to high wear due to metal-on-metal articulation are likely to be similar in hip resurfacing as well as large-diameter hip arthroplasty [1]. Moroni et al. [4] also found no differences in mean Co, Cr and Mo levels between patients with metal-on-metal hip resurfacing and metal-on-metal THA at 2- and 5-year follow-up. Furthermore, there were no significant differences within the implant groups at each time of determination. Only the ion concentrations were greater in patients with metal-on-metal devices compared to a control group (p < 0,001)[4].

Otherwise, the current study and even the studies of Garbuz et al.[3] and Vendittoli et al. [28] showed that patients with metal-on-metal total hip arthroplasties had significantly higher concentrations of blood metal ions compared to patients undergoing hip resurfacing (Table 2).

The variability of the serum levels of metal ions is influenced by several factors such as different implant size, incorrect adjustment of the components, differences in manufacture and metallurgy as well as metal corrosion [4-8,16,17,21,22]. Shimmin et al. [21] reported the results of the Australian National Joint Registry showing an inverse relationship between the size of the femoral component and the risk of revision (component size <44 mm have a fivefold higher risk of revision) following hip resurfacing. In the series of Langton et al. [7], 17 patients with the ASR^TM^ device were revised due to adverse reaction to metal debris (ARMD). This group had significantly smaller components, higher acetabular component anteversion and higher whole blood concentrations of Co and Cr in contrast to asymptomatic patients (all p < 0,001).

In several other studies increased components' wear, edge loading and raised blood and/or serum ion levels were also correlated positively with the inclination of the acetabular component [1,5-8,16,17,21,22]. A comparative study of Langton et al. [17] showed increased metal ion levels in patients with resurfacing devices and an inclination greater than 45°, while Witzleb et al. [14] showed inverse results. Furthermore, a significant inverse relationship could be found between femoral size and Co and Cr concentrations [17]. Therefore, the authors stated that larger resurfacing implants seem to be more resistant to suboptimal position in terms of metal ion generation. This might be due to the thicker fluid film as well as the greater arc of cover [17]. On the other hand, the recent study showed no correlation between the metal ion levels in the serum, the inclination of the acetabular component, and the arc of cover (Table 3), possibly due to the small number of patients included in the study.

This study has some limitations: First, we only observed metal ion levels after a minimum follow-up of 2 years and therefore lack information of possible long-term effects and outcome. Second, the radiological evaluation of inclination and arc of cover lacks inter- and intraobserver coefficients. Third we observed a relatively small number of patients and the patients enrolled are split into two subpopulations. Fourth we did not perform an a priori power analysis but differences of outcome between the THA group compared to the resurfacing group were large enough yielding statistical significance and therefore adequate post hoc power.

## Conclusion

Determining the serum concentrations of metal ions in patients following metal-on-metal hip resurfacing as well as metal-on-metal THA showed increments for Co and Cr. Furthermore, patients treated with large-diameter metal-on-metal total hip prostheses showed threefold and fourfold, respectively, higher levels for Co and Cr compared to patients with metal-on-metal resurfacing devices, 2-years following implantation. The effects of systemic metal ion exposure in patients with implants made of Co and Cr continue to be a matter of concern. In order to clarify the biologic effects of ion dissemination and to identify risks concerning long-term toxicity of metals, the exposure should be monitored carefully. Therefore, long-term studies have to be done to determine adverse effects of Co and Cr following metal-on-metal hip replacement.

## Competing interests

The authors declare that they have no competing interests.

## Authors' contributions

WME, JF, MP and MT participated in the design and the coordination of the study. WME, JF, PS and AL performed acquisition of the data. WME, JF, MP and PS drafted the manuscript. AL reviewed the manuscript critically. All authors read and approved the final manuscript.

## Pre-publication history

The pre-publication history for this paper can be accessed here:

http://www.biomedcentral.com/1471-2474/13/56/prepub
